# A unique insert in the genomes of high-risk human papillomaviruses with a predicted dual role in conferring oncogenic risk

**DOI:** 10.12688/f1000research.19590.2

**Published:** 2019-10-01

**Authors:** Noam Auslander, Yuri I. Wolf, Svetlana A. Shabalina, Eugene V. Koonin

**Affiliations:** 1 National Center for Biotechnology Information, National Institutes of Health, USA, Bethesda, Maryland, 20814, USA

**Keywords:** Papillomaviruses, cervical cancer, oncogenic risk, translation terminatio-reinitiation, machine learning

## Abstract

The differences between high risk and low risk human papillomaviruses (HR-HPV and LR-HPV, respectively) that contribute to the tumorigenic potential of HR-HPV are not well understood but can be expected to involve the HPV oncoproteins, E6 and E7. We combine genome comparison and machine learning techniques to identify a previously unnoticed insert near the 3’-end of the E6 oncoprotein gene that is unique to HR-HPV. Analysis of the insert sequence suggests that it exerts a dual effect, by creating a PDZ domain-binding motif at the C-terminus of E6, as well as eliminating the overlap between the E6 and E7 coding regions in HR-HPV. We show that, as a result, the insert might enable coupled termination-reinitiation of the E6 and E7 genes, supported by motifs complementary to the human 18S rRNA. We hypothesize that the added functionality of E6 and positive regulation of E7 expression jointly account for the tumorigenic potential of HR-HPV.

## Introduction

Persistent infections with carcinogenic human papillomaviruses (HPVs) are the main cause of cervical neoplasia and cancer, with over 99% of the cervical lesions containing HPV sequences ^1– 3^. There are currently 198 HPV types that have been officially numbered ^4^, of which approximately a third are predominantly detected in the cervical epithelium and belong to the *Alphapapillomavirus* genus ^5, 6^. The viruses of this genus are further grouped into high-risk (HR) and low-risk (LR) HPV types based on their association with cervical cancer and pre-cancerous lesions ^7, 8^. Most of the HR-HPV variants belong to the *Human papillomavirus 16* (alpha-9) or *Human papillomavirus 18* (alpha-7) species groups ^9^.

Phylogenetic trees constructed from alignments of complete HPV genomes cluster all oncogenic types together, suggesting a common ancestor for the HR-HPVs. However, in separate trees built from different regions of the genome, the carcinogenic potential co-segregates with the early (those produced prior to replication) but not with the late genes ^10, 11^. The early HPV proteins E6 and E7 have transforming properties ^12– 14^ and are required for the malignant conversion. The involvement of these proteins in tumorigenesis is thought to stem from their interactions with the tumor suppressors p53 and pRB, respectively, as well as other proteins involved in tumorigenesis ^14– 20^. Variations in E6 and E7 proteins have been suggested to determine the oncogenic potential of HPV ^21, 22^ but the potential discriminating features of the oncogenic variants are frequently observed in LR-HPVs as well ^23– 27^. A notable molecular feature that distinguishes HR from LR-HPV (along with pronounced sequence differences) is the presence of a PDZ-domain recognition motif at the extreme C terminus of the HR-E6 oncoprotein, as opposed to LR-E6 ^28– 30^, which enables interactions of HR-E6 with numerous PDZ domain proteins ^31– 33^.

Both oncogenes are transcribed from an early promoter as a single E6-E7 polycystronic pre-mRNA. The transcriptional level and translational efficacy of this RNA are regulated by the alternative RNA splicing machinery of host cells ^34, 35^. Alternative splicing of introns in the E6 gene produces multiple splice isoforms of E6-E7 pre-mRNA ^34,36^. In HR-HPV16 and HPV18, E6*I is one of the major splice isoforms that functions as the mRNA for the production of E7 via translation reinitiation ^37^, In contrast, unspliced E6 mRNA is responsible for the production of E6 ^34, 37^. Another recent study has shown that HPVs generate single-stranded circular RNAs (circRNAs), some of which encompass the E7 gene (circE7) ^38^.

Identification of signatures of the HR-HPV genotypes that differentiate them from the majority of alpha papillomaviruses that lack oncogenic potential could help elucidate the genetic basis of the carcinogenic properties of HPVs, thus contributing to a better understanding of the biological mechanisms exploited by the virus to trigger neoplasia. However, at present, genomic determinants of the HPV oncogenic risk are not well understood, and the exact nature of the genetic changes that led to the emergence of the HR-HPV oncogenicity remains unknown.

To better understand HPV carcinogenesis, we revisited the search for specific genomic determinants of HR-HPV types and identified a previously unreported insert of 30 to 60 base pairs (bp) at the 3’-end of the E6 oncoprotein coding region that is present in all HR-HPV types but not in LR-HPV. This insert introduces a new stop codon, separating the nucleotide sequence coding for E6 from that coding for E7, thus, eliminating the overlap between E6 and E7 that is characteristic of the LR-HPV types. The insert confers a PDZ binding motif at the end of E6 oncoprotein which is likely important for the oncogenic potentiation. Additionally, the insert places the termination codon of E6 upstream but in close proximity of the initiation codon of E7. Furthermore, the insert contains sequences complementary to human 18S rRNA in the regions of hairpins 26 and 27 that are known to interact with viral RNAs and are specifically involved in IRES (Internal Ribosomal Entry Sites) binding and cap-independent translation ^39– 42^. We hypothesize that the insert separating the coding sequence of E6 and E7 was the primary cause of the emergence of high oncogenic potential alpha-HPV.

## Results

**Figure 1.**
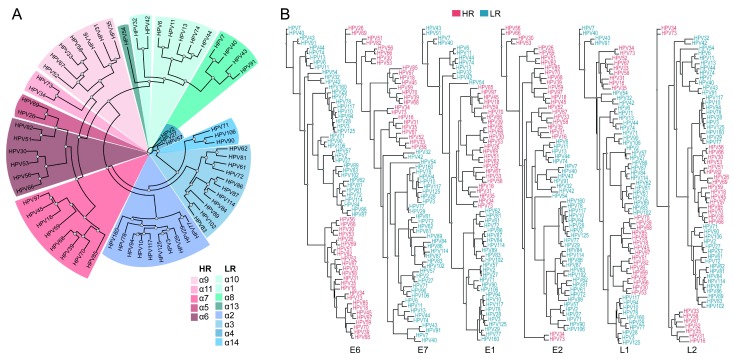
Phylogenetic trees of alpha-HPV. ( **A**) Maximum likelihood tree obtained with the whole genome nucleotide sequences alignment of alpha-HPV, colored by the different alpha-class categories. ( **B**) Maximum likelihood trees obtained with alignments of E6, E7, E1, E2, L1 and L2 amino acid sequences of alpha-HPV, colored by the oncogenic risk groups.

The complete genome sequences and amino acid sequences of HPV E1, E2, E6, E7, L1 and L2 proteins were collected for all sequenced alpha-HPV strains ( **pave.niaid.nih.gov** ^43^). We then constructed a global multiple sequence alignment of the whole genome nucleotide sequences and the amino acid sequences alignments for each protein. Maximum likelihood phylogenetic analysis of these alpha-HPVs, based on the complete nucleotide sequence, as well as the amino acid sequences of most of the early proteins, identified HR-HPV as a clade, whereas phylogenies of L1 and L2 did not support the monophyly of the HR-HPV ( Figure 1), in agreement with previous findings ^9, 10^. These observations are compatible with a major role of recombination in HPV evolution.

We next sought to identify genomic features that might partition alpha-HPV species in accord with their oncogenic risk, focusing on E6 and E7 oncogenes.

**Figure 2.**
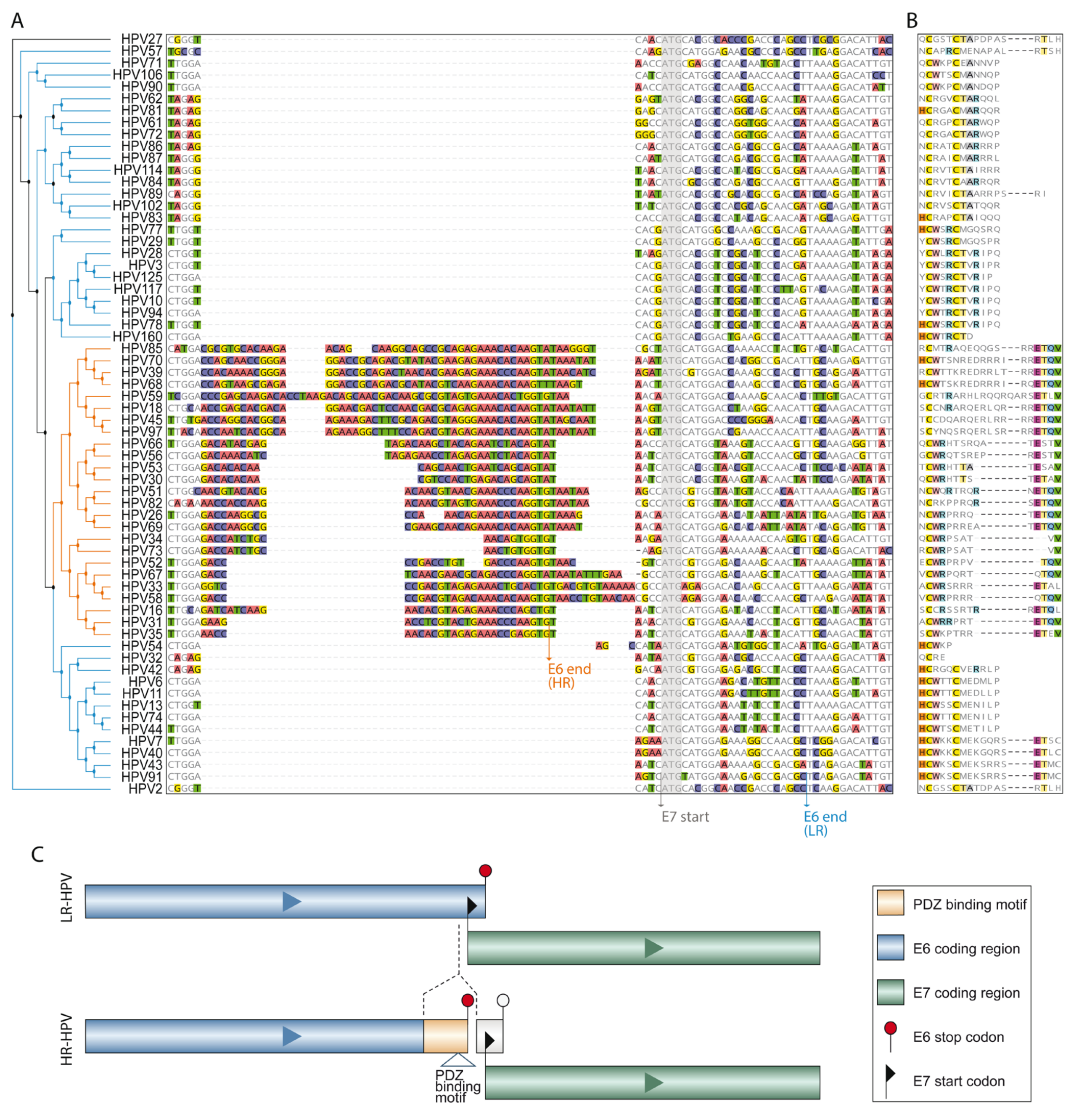
HR-HPV-specific sequence insert. ( **A**) Nucleotide sequence alignment of alpha-HPVs (blue, LR-HPV; orange, HR-HPV sequences). Arrows: Grey, E7 start codon for most HPV types; Blue and Orange, E6 stop codons that are distinct between LR-HPV and HR-HPV. ( **B**) Alignment of the C-terminal amino acid sequences of E6 proteins of alpha-HPVs. ( **C**) Schematic representation of the E6/E7 separation caused by the insertion in the 3’-terminal region of E6 and the proximity of E6 stop and E7 starts in HR-HPV.

First, we searched for regions of insertions and deletions within the genome nucleotide sequences of E6 and E7 that might differentiate between the risk groups. Specifically, we identified sequences with high frequency of deletions or insertions that are located within high confidence alignment regions (See *Methods* for details). We then applied Support Vector Machine (SVM), a linear classification technique, with a leave-one-out cross-validation, aiming to identify regions that differentiate between high-risk and low-risk strains. This approach resulted in the identification of genomic regions that separated HR-HPV from LR-HPV with high accuracy (>0.75, with statistical significance; see *Methods*). Among these, we found one prominent insert (between 30 and 60 nucleotides) located in the 3’-terminal region of the E6 gene ( Figure 2A). We also performed a similar search for regions separating HR-HPV from LR-HPV, using the amino acid sequence of E6 and E7 oncoproteins. For the purpose of classification, we coded the amino acids with numbers based on their frequencies and the BLOSUM62 ^44^ matrix (see *Methods*). As expected, the divergent region in E6 was identified from the amino acid sequences as well ( Figure 2B). In contrast, in the E7 protein sequences, we did not find any significant differences between the high risk and low risk HPV strains ( *Extended data:* Figure S2 ^45^).

The discriminating region identified in the E6 gene contains an insert with a conserved sequence in all HR-HPV strains. The insert contains an in-frame stop codon for the E6 coding sequence, which eliminates the overlap between the coding sequences of E6 and E7 that is characteristic of the LR-HPV genomes, but results in nearly identical lengths of the E6 proteins in HR and LR-HPV strains albeit with unrelated C-terminal sequences of 8-19 amino acids ( Figure 2B). The unique C-terminal sequence of HR-HPV E6 that originates from the insert contains a PDZ domain-binding motif X-T-X-V/L at the very C-terminus of E6 in almost all HR-HPVs. Indeed, several PDZ domain-containing proteins have been identified as binding partners of HR-E6, including hDlg, hScrib, MAGI-1, MAGI-2, MAGI-3, and MUPP1 ^29, 32, 33, 46– 48^. These interactions that are unique for HR-HPV are likely to contribute to HR-HPV induced oncogenesis ^49^.

We observed that the sequence similarity between the insert sequences among HR-HPV strains is more pronounced at the nucleotide level than at the amino acid level (See METHODS for details and *Extended data:* Figure S3 ^45^). Combined with the separation between the coding regions of E6 and E7 resulting from the insertion, and the proximity of E6 stop codon to the E7 start codon, this finding led us to hypothesize that the insert has an additional role as a regulatory element. Furthermore, as E7 has been previously identified as the dominant oncogene ^50^, the lack of genomic determinants of HR-HPV within the E7 gene is compatible with the possibility that the insert contains regulatory elements enhancing E7 expression in HR-HPV strains.

**Figure 3.**
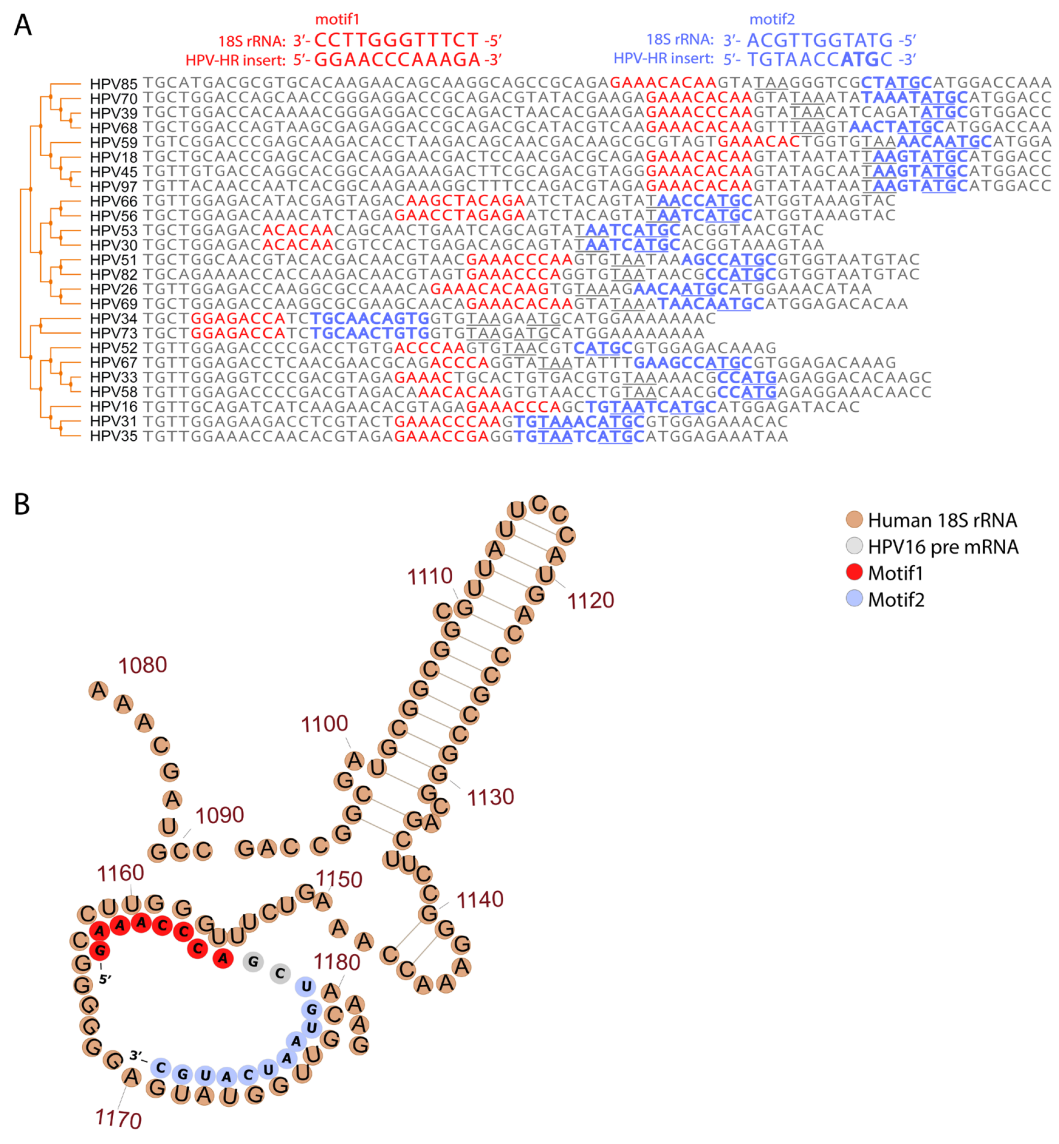
Sequences complementary to 18S rRNA sequence in the HR-HPV-specific insert. ( **A**) Comparison of 18S rRNA complementary sequences for different HR-HPV strains. The distal and proximal to the E7 start motifs (motifs 1 and 2) to the E7 start codon are shown in red and blue, respectively. The E6 stop codons (TAA) and E7 start codons are marked in bold. ( **B**) An illustration of the potential interactions between folded HPV16 pre-mRNA (grey, red and blue) and the human 18S rRNA (brown). The two motifs are marked on the HPV-structure in red (motif 1) and blue (motif 2).

Several cases of coupled termination-reinitiation for polycistronic mRNA with proximate stop and start codons are evident for translation of eukaryotic virus genes ^51– 57^. The efficiency of this process depends on the close proximity of the termination and reinitiation sites ^57, 58^, and the presence of motifs complementary to the 18S rRNA in the mRNA sequence ^51, 52, 57, 59^. Given the proximity of the E6 termination site to the E7 initiation site codon that results from the insertion in the HR-HPV strains conferred by the insert, we investigated the possibility of coupled termination-reinitiaion of E6 and E7 in these strains. Notably, within the inserted sequence in the vicinity of the E7 start codon, we identified two conserved regions that are complementary to the sequences in 18S rRNA hairpins 26 and 27 which are commonly involved in the interactions between ribosomes and virus IRES and responsible for cap-independent translation ^60^ ( Figure 3A). The recognition by host 18S rRNA was shown to be important factor for active termination-reinitiation for polycistronic mRNA with proximate stop and start codons ^51, 52, 57, 59^. The first region of complementarity consistently forms an internal loop and a relaxed, unpaired structure in the predicted optimal and sub-optimal E6-E7 mRNA folding of HR-HPV strains ^58^ ( *Extended data:* Figure S4 ^45^ , see Methods). The second region of 18S RNA complementarity overlaps with the E7 start site, and hence might function independently or cooperate in the regulation of E7 translation ( Figure 3B). Also, these regions of complementarity might be important for generation of circE7 and for translation regulation of E7 encoded by circE7 or by alternative transcripts through cap-independent mechanisms. These findings suggest that the insertion could enable coupled termination-reinitiation of E6 and E7 proteins and could regulate E7 translation encoded by E6E7 alternative transcripts and circE7, enhancing their combined expression in HR-HPV, and thus promoting the oncogenic transformation induced by these viruses.

Furthermore, it has been recently reported that oncogenic human HPVs generate single-stranded circular RNAs (circRNAs), some of which encompass the E7 gene (circE7) ^38^. CircE7 is represented in the TCGA RNA-Seq data from HPV-positive cancers, and specific disruption of circE7 in CaSki cervical carcinoma cells reduces E7 protein levels and inhibits cancer cell growth both *in vitro* and in tumor xenografts ^38^. Given that the insert identified here is located in the 5’UTR of cicrE7 and in the proximity of the circE7 backsplice junction, it might increase the E7 translation efficacy from circE7 as well as facilitate the generation of the circE7 RNA.

## Discussion

The genus *Alphapapillomavirus* includes HPV types that are uniquely tumorigenic. However, the events in the HPV genome evolution that led to the carcinogenic potential of some alpha-HPV types remain poorly understood. Here, we revisited this problem by performing a search for genomic determinants of the oncogenic risk of alpha-HPV types and identified a unique insert in the 3’-terminal regions of the E6 oncoprotein genes in all HR-HPV strains. To the best of our knowledge, this insert in HR-HPV genomes has not been reported previously. The insertion maintains closely similar lengths of E6 proteins in HR-HPV and LR-HPV types, which could explain why it has been overlooked in previous HPV genome analyses.

We hypothesize that the insertion makes a dual contribution to the oncogenicity of the HR-HPV types. First, the inserted sequence changes the C-terminal amino acid sequence of E6 and creates a PDZ domain-binding motif that is unique to HR-HPV types. The experimentally demonstrated interaction between the E6 proteins of HR-HPV and several PDZ domain-containing proteins is thought to contribute to HPV-induced tumorigenesis ^30, 32, 61^. Interestingly, PDZ-binding motifs have been identified also in several other oncogenic viruses, such as HTLV-1, adenovirus RhPV1 and beta-HPV8 ^62, 63^. Second, the insert eliminates the overlap between the E6 and E7 coding regions, implying a possible role as a regulatory element. We found that almost all HR-HPV genomes contain the sequence T-A/G-T-A-A-T/A in the insert near the end of the E6 coding sequence, which is closely similar to the sequence of the early promoter at the 5’ end of E6 that is employed for the synthesis of the E6-E7 mRNA ^64, 65^. However, for the HR-HPV strains, unlike the case of the LR-HPV ^66^, there are no reports of an independent E7 promoter, so that E6 and E7 are both translated from a polycistronic mRNA. Thus, the promoter-like sequence within the insert is likely to be spurious.

In HR-HPV strains, E6 and E7 proteins are translated from a polycistronic pre-mRNA ^67^, and translation reinitiation has been suggested as the mechanism ^37, 66, 68^. However, the close proximity of the E6 stop codon to the E7 start codon in HR-HPV (only a few nucleotides separating these codons) could inhibit re-initiation ^66, 68^. Therefore, it has been suggested that ribosomal reinitiation is enabled through the formation of the E6*I splice variant (removal of intron I) in which the intercistronic distance between the translation termination codon of E6*I and the E7 initiation codon is increased ^37^. Thus, E7 protein translation might be enhanced by the removal of the intron I region from pre-mRNAs by splicing, whereas retention of that sequence is required for E6 translation ^69^. A comprehensive characterization of HPV16 and HPV18 expression by RNA-Seq analysis in invasive cervical cancer has shown that E6*I is the most abundant transcript isoform for both viral types ^70^. The insert identified here is located in the 5’UTR immediately upstream of the E7 start codon in the E6*I transcript, and there are two alternative branch sites for E6*I splicing to produce a E7 mRNA with variable 5’UTRs ^71^. However, several studies have reported that E7 translation is independent of splicing within the E6 open reading frame ^68, 72, 73^ , undermining this interpretation.

Further investigating the potential regulatory role of the inserted sequence, we identified two conserved motifs that are complementary to the human 18S rRNA ( Figure 3); interaction of such motifs with the rRNA has been shown to play a role in coupled termination-reinitiation for several viral genes ^51, 52, 57, 59^. The first motif forms an internal loop in the predicted mRNA secondary structure of E6-E7, whereas the second one overlaps with the E7 initiation codon. Given the evolutionary conservation of these motifs and the close proximity of E6 termination site to the E7 initiation site, it appears plausible that coupled termination-reinitiation promoted by the insert sequence is an important mechanism for E7 translation in HR-HPV strains.

The folding of these regions of rRNA complementarity in E6-E7 mRNAs is typically relaxed in the predicted optimal and sub-optimal secondary structures of the HR-HPV strains. The insert is present not only in polycistronic *E6E7* pre-mRNAs, but also in some alternatively spliced *E6E7* transcripts, including the most common spliced isoform E6*I in HR- HPV16 and HPV18 ^70^. The biologically functional circE7 that has been identified in TCGA RNA-Seq data from HPV-positive cancers ^38^, contains the unique insert in its 5’UTR. Thus, the regions of complementarity to human 18S rRNA could modulate the translation rate of the E7 encoded by alternative transcripts and circRNAs in HR-HPV.

Recent analysis of 5570 cervical HPV16 genomes ^74^, in which the E6 gene was found to be highly variable whereas the E7 gene was strikingly devoid of genetic variants in precancer and cancer cases, support the notion that idea that E7 is a the contributor in HR-HPV-induced carcinogenesis ^50, 75^. Thus, enhancement of E7 production by regulatory elements contained in the insert is likely to substantially affect the oncogenicity of the HR-HPV strains ^76^. The insert is likely to enable coupled termination-reinitiation of the E6 and E7 genes, which is supported by the presence, within the insert sequence, of motifs complementary to the human 18S rRNA. In addition to the enhancement of reinitiation during the E6-E7 mRNA translation, this interaction might affect the dynamics and efficiency of E7 translation from alternative transcripts in HR-HPV. Furthermore, these motifs might also regulate E7 translation encoded by circE7 through cap-independent mechanisms. The dynamics and the rate of translation of E7 alternative isoforms appear to vary and seem to influence the stability of E7 proteins differentially across HPV types ^34, 70, 76^. Thus, potential 18S rRNA – insert duplexes could modulate the rate of E7 translation initiation and elongation in HR-HPV and result in differences in stability of HR-HPV E7 proteins compared to LR-HPV, and thus could affect the interactions between E7 and various protein partners.

Given the lack of additional major genomic determinants consistently differentiating between HR-HPV and LR-HPV, it seems most likely that the insert in the E6-E7 transcript is the primary cause behind the emergence of oncogenic HPV and makes a complex contribution to the oncogenicity of the HR-HPV types in which both E6 and E7 oncoproteins are involved.

## Methods

### Multiple sequence alignment and phylogenetic analysis

Multiple alignments of nucleotide and amino acid sequences that were obtained from PaVE (pave.niaid.nih.gov ^43^) were generated using MAFFT v7.407 ^77^ with default parameters. Maximum likelihood phylogenetic analysis was performed using the resulting alignments and PhyML 3.1 software ^78^, with the Bayesian information criterion, NNI tree improvement and an LRT SH-like likelihood method for support estimation.

### SVM applied to nucleotide sequences

To apply Support Vector Machines (SVM ^79^, using Matlab 2018b *fitsvm* function) to the nucleotide sequences, we first encoded the data with numbers, where each nucleotide is coded as ‘1’ and each gap as ‘0’. We searched for alignment regions with deletions or insertions in multiple HPV strains that are surrounded by high confidence alignment regions (alignment regions of length > 15 bp containing less than 5% of gaps in each position) because these are most likely to contain relevant differences within conserved genomic regions. Within these regions, we then trained the SVM to classify alpha-HPV strains based on their oncogenic potential. The performance of the SVM was evaluated by leave-one-out cross validation, and regions with the overall balanced accuracy >0.75 (the average of the accuracy for positive and negative classes) were selected for further analysis.

### SVM applied to amino acid sequences

To apply SVM (using Matlab 2018b *fitsvm* function) to the amino acid sequences of E6 and E7 proteins, we first encoded the amino acids with numbers using the frequencies of each amino acid in each protein and the BLOSUM62 matrix ^44^. For each position, the most frequent amino acid was identified, and the amino acids in each protein were encoded by their BLOSUM62 distances from the most frequent amino acid in the respective position. We then trained the SVM to classify alpha-HPV strains based on their oncogenic potential using the coded protein sequences. Positions surrounded by high-confidence alignment regions (length > 5 amino acids and containing less than 5% of gaps in each position) were selected for further analysis. For these positions, we evaluated the performance by leave-one-out cross validation, and regions with the overall balanced accuracy >0.75 were selected for further estimation of significance using a permutation test.

### Estimation of the significance of the identified regions using permutations

To estimate the significance of the identified regions, i.e. to determine whether similar differences could be observed by chance, we performed a permutation test ( *Extended data* ^62^), controlling for the topology of the reconstructed phylogenetic trees. To this end, the labels were randomly permuted while maintaining unified labels for clades with high similarity. For each identified region, the likelihood of obtaining an equivalent or higher performance for the length of the region within the respective protein was evaluated by calculating an empirical P-value. We consider regions with permutation P-value <0.05.

### Analysis of RNA folding and RNA-RNA duplexes

The most stable secondary structures were predicted for all HR-HPV strains and their free energy values were calculated using the Vienna package ^60^. Afold and Mfold were applied for prediction of optimal and sub-optimal mRNA structures ^80, 81^. Target opening free energy was estimated for motifs 1 and 2 using optimal and sub-optimal RNA structures, as described previously ^82^. The sequence fold variants with the lowest secondary-structure free energy are presented in the *Extended data:* Figure S4 ^45^. The formation of intermolecular mRNA-rRNA duplexes and hybridization affinity of the E6-E7 inserts to ribosomal RNA were evaluated using the Hybrid software with default parameters, with a ΔG threshold of ≤–10 kcal/mol ^83, 84^. The human 18S rRNA 2D structure was downloaded from http://apollo.chemistry.gatech.edu/RibosomeGallery/;.

## Data availability

### Underlying data

Nucleotide sequences of HPV and amino acid sequences of HPV E6 and E7 proteins are available from PaVE database **pave.niaid.nih.gov** ^43^

### Extended data

Harvard Dataverse: A unique insert in the genomes of in high-risk human papillomaviruses with a predicted dual role in conferring oncogenic risk, https://doi.org/10.7910/DVN/FUGEUB ^45^.

This project contains the following extended data:

• Table 1: Complete nucleotide sequences and the amino acid sequences of HPV E1, E2, E4, E5, E6, E7, L1 and L2 proteins. This is the only source data that was required and employed for the analysis reported in this work.
• Figure S1: Maximum likelihood trees obtained with alignments of E6, E7, E1, E2, E4, E5, L1 and L2 amino acid sequences of alpha-HPV strains.
• Figure S2: The balanced accuracy (y-axis) obtained from a leave-one-out cross validation for predicting risk category (HR vs LR) of alpha-HPV strains using BLOSUM62 coding of amino acid sequences, of different positions (x-axis) E6 (A) and E7 (B). Zero-accuracy was assigned to regions surrounded with low confidence alignment.
• Figure S3: Boxplots showing the distributions of the identity fraction of each nucleotide (NN) and amino acid (AA) in the genome and protein sequences of the insertion (not considering gaps for both NN and AA). The individual identity fractions of each position are overlaid.
• Figure S4: RNA fold secondary structure prediction of HR HPV strains 16 (A), 18 (B), 45 (C) and 31 (D). The nucleotides of the first motif are marked in red, and of the second motif in purple. E7 AUG is noted in red font.

Data are available under the terms of the Creative Commons Zero "No rights reserved" data waiver (CC0 1.0 Public domain dedication).

Zenodo: Permutation test controlling for HPV strains, http://doi.org/10.5281/zenodo.3242231 ^85^. Apache License, Version 2.0.
