# Supplementary material for: A unique insert in the genomes of high-risk human papillomaviruses with a predicted dual role in conferring oncogenic risk
Source: F1000Res. 2019 Oct 1;8:1000. Originally published 2019 Jul 2. [Version 2] doi: 10.12688/f1000research.19590.2 (PMC6685453; doi:10.12688/f1000research.19590.2)
Supplement: Peer review history

# Peer review: Reviewer response for version 2

**DOI:** 10.5256/f1000research.22707.r54507

**Published:** 2019-10-16

**Authors:** Alison McBride

**Affiliations:** 1 DNA Tumor Virus Section, National Institute of Allergy and Infectious Diseases (NIAID), Bethesda, MD, USA

I confirm that I have read this submission and believe that I have an appropriate level of expertise to confirm that it is of an acceptable scientific standard.

# Peer review: Reviewer response for version 2

**DOI:** 10.5256/f1000research.22707.r54508

**Published:** 2019-10-08

**Authors:** Elizabeth A. White

**Affiliations:** 1 Department of Otorhinolaryngology: Head and Neck Surgery, University of Pennsylvania Perelman School of Medicine, Philadelphia, USA

I approve the manuscript in its revised form.

I confirm that I have read this submission and believe that I have an appropriate level of expertise to confirm that it is of an acceptable scientific standard.

# Peer review: Reviewer response for version 1

**DOI:** 10.5256/f1000research.21480.r51570

**Published:** 2019-08-06

**Authors:** Elizabeth A. White

**Affiliations:** 1 Department of Otorhinolaryngology: Head and Neck Surgery, University of Pennsylvania Perelman School of Medicine, Philadelphia, USA

In this study, Auslander and colleagues compare nucleotide and amino acid sequences for a subset of genus alpha HPV genotypes. They observe that high-risk HPV genomes share a short sequence insert at the 3’ end of the E6 ORF. The insertion adds a PDZ binding motif to the C-terminus of high-risk HPV E6 proteins and alters the location of the E6 termination codon relative to the E7 initiation codon.

 This observation is consistent with previous findings. It has been appreciated for some time that high-risk HPV E6 and E7 are transcribed from a polycistronic mRNA whereas low-risk E6 and E7 are transcribed from separate promoters. In addition, a frequent splicing event occurs within the E6 gene in the bicistronic high-risk HPV early mRNA. This report adds to those observations. It proposes that the 3’ high-risk specific insert is another feature that differs between high- and low-risk HPV and that it might drive differences in expression of high-risk versus low-risk HPV oncoproteins. Understanding the differences between oncogenic and non-oncogenic HPV is an area of intensive research and new contributions in this area are potentially significant. This report makes a useful contribution to the literature.

 Weaknesses of the manuscript are that the current literature is not cited and that other features of high-risk HPV E6 that might also account for their oncogenic activities are not discussed. Although comparing nucleotide sequence differences is informative, for HPV E6 this comparison does not completely reflect the biology of the proteins. There are high-risk HPV E6-specific protein binding partners other than PDZ domain proteins. Several of these are listed in a useful 2012 review ^1^; others were identified by proteomic analyses from several groups ^2^ ^3^. For example, the authors do not mention that TP53 binding and degradation is a feature of high-risk HPV E6 not shared by low-risk HPV E6. It also appears that not all of the high-risk HPV E6 interact with the same subset of cellular PDZ proteins. It is unclear how these diverse HPV E6-PDZ protein interactions might account for the shared oncogenic features of high-risk HPV E6, and this point is not discussed in the manuscript.

 It is established in the HPV literature that small differences in HPV oncoprotein amino acid sequence result in significant differences in interactions with host cellular proteins. This is beautifully illustrated by the structural studies of Gilles Trave and colleagues, who have determined that subtle differences in E6 enable a range of specific interactions with cellular LxxLL-containing proteins. Other recent studies highlight high-risk HPV-specific activities of the E7 oncoprotein. In light of findings like these, this manuscript seems to overstate the claim that the PDZ binding motif is the ‘most notable molecular feature’ distinguishing high- from low-risk HPV. The potential importance of the insert sequence to E6/E7 translation regulation is high and should be tested; the discussion of the importance of the PDZ binding motif should be tempered and put in context with other recent findings.

 Additional points:

1. Mirabello and colleagues recently reported an analysis of sequence variants from >5000 HPV16-positive cervical samples ^4^. HPV16 E6 sequences exhibited variation across the length of the ORF that was similar in high-grade versus control lesions. A possible interpretation of this finding is that the protein sequence in the C-terminus of E6 is not important for oncogenic transformation. How does this fit into the authors’ model? It would be useful to include a discussion of these data.
2. The phylogenetic trees in Figure 1B might be easier to interpret if they were presented in a linear format. It is difficult to determine where the boundaries between the high-risk/low-risk groupings overlap with the major branch points of the tree.
3. HPV diversity is much greater that that reflected solely in genus alpha. It would be interesting to know whether HPV from other genera have also acquired sequences in this region that might provide some or all of the activities suggested by the authors.

I confirm that I have read this submission and believe that I have an appropriate level of expertise to confirm that it is of an acceptable scientific standard.

## References

[1] : Cellular transformation by human papillomaviruses: lessons learned by comparing high- and low-risk viruses. *Virology*.2012;424(2) : 10.1016/j.virol.2011.12.018 77-98 10.1016/j.virol.2011.12.018 22284986PMC3703738

[2] : Interpreting cancer genomes using systematic host network perturbations by tumour virus proteins. *Nature*.2012;487(7408) : 10.1038/nature11288 491-5 10.1038/nature11288 22810586PMC3408847

[3] : Comprehensive analysis of host cellular interactions with human papillomavirus E6 proteins identifies new E6 binding partners and reflects viral diversity. *J Virol*.2012;86(24) : 10.1128/JVI.02172-12 13174-86 10.1128/JVI.02172-12 23015706PMC3503137

[4] : HPV16 E7 Genetic Conservation Is Critical to Carcinogenesis. *Cell*.2017;170(6) : 10.1016/j.cell.2017.08.001 1164-1174.e6 10.1016/j.cell.2017.08.001 28886384PMC5674785

# Response

**Published:** 2019-09-13

**Authors:** Eugene Koonin

**Affiliations:** National Center for Biotechnology Information (NCBI), National Institutes of Health (NIH), USA

We appreciate this highly constructive and helpful review in response to which the following modifications have been made too the article:

1. Many references to the current literature were added, and several other features of HR-HPV E6 are now discussed.
2. The suggested references to HR-HPV E6 specific binding partners have been included.
3. The discussion on the importance of the PDZ binding motif in distinguishing HR from LR HPV has been tempered, and the context for its potential relevance has been clarified.
4. We now discuss the recent analysis of 5570 cervical HPV16 genomes (Mirabello and colleagues) and have substantially expanded the discussion of the effect of the insert on E7 production.
5. Figure 1B has been updated, phylogenetic trees are now presented in a linear format.

​​​​​​​In addition, although the referee have suggested a statistical review of the permutation test that was used in this work to assess the significance of the results, we are confident that this is unnecessary because the statistical technique we used is simple and standard.               

# Peer review: Reviewer response for version 1

**DOI:** 10.5256/f1000research.21480.r51173

**Published:** 2019-07-31

**Authors:** Alison McBride

**Affiliations:** 1 DNA Tumor Virus Section, National Institute of Allergy and Infectious Diseases (NIAID), Bethesda, MD, USA

Auslander and colleagues present a comparative sequence analysis of alpha-papillomavirus genomes. They identify and analyze a short 30-60 nucleotide sequence insert between the E6 and E7 open-reading frames that encodes the PDZ domain in the high-oncogenic risk types and propose that this region also contains sequences that enable coupled termination and reinitiation to facilitate translation of the E6 and E7 proteins. This is an intriguing observation as HPVs are associated with ~5% human cancers and understanding how the viral oncoproteins are expressed is crucial. The proposed hypothesis should be testable and indicates that experiments in which the E6 PDZ domain is mutated in the background of the viral genome should be interpreted carefully.

 A weakness of the manuscript is that current literature and data sources are not used/and or cited and the data-set used seems incomplete. For example, there are currently 198 officially numbered HPV types, and 442 HPV types in total. A curated data-set of genome sequences for all papillomavirus types sequenced to date is available at PaVE. There are also many recent publications that describe analyses of PV evolution, oncogenicity and E6 PDZ domains that should be cited.

 There are sequences available for 64 alpha HPV types that have been officially named by the HPV Reference Centre, yet only the genomic sequences of 44 types are listed in Figure S1. The data-set contains many isolates for some HPV types (e.g. 299 isolates of HPV16) yet almost no representatives of the species alpha 2, 4 and 8. The E6 protein of HPV40 (alpha-8) has been proposed to contain an ancestral alpha PDZ domain and so the genomes of the alpha-8 species should be closely examined/discussed. Nevertheless, a nucleotide alignment of all 64 alpha-PV nucleotide sequences from PaVE does support the authors’ conclusions that only HR species-5, 6 7, 9 and 11 contain an insert separating the E6 and E7 ORFs.

**Minor issues:**

1. In Figure 1A, alpha-11 is listed in the key for both LR and HR.
2. In Figure 2, the resolution of the sequence images should be improved. The legend for 2B should make it clear that these are just the C-terminal sequences of the E6 polypeptides. In 2C, the symbols and colored blocks should be described.
3. In Figure 3A, the color/bold identification of the motifs and TAA/AUG are not easily visualized in the pdf. Perhaps highlight TAA/AUG by underlines or boxes. Labelling the groups of different HPV species along the left would also be helpful. In 3B, the labelling of the 18S rRNA hairpin (orange text) is confusing. What is 26es7?
4. In Figure S4: clarify in the legend that this is the E6-E7 RNA sequence for each HPV type shown.
5. The reference for IRES (54), I think should be 53.

I confirm that I have read this submission and believe that I have an appropriate level of expertise to confirm that it is of an acceptable scientific standard.

# Response

**Published:** 2019-09-13

**Authors:** Eugene Koonin

**Affiliations:** National Center for Biotechnology Information (NCBI), National Institutes of Health (NIH), USA

We appreciate the constructive and very helpful review in response to which the following changes have been made to the manuscript:

 

1. We now mention the most recent number of  HPV types that have been formally recognized (198), and the reference supporting this has been updated.
2. We added numerous references to recent publications that describe analyses of HPV evolution, oncogenicity, oncoprotein interactions and E6 interactions with PDZ domains.
3. We now include all 64 alpha HPV types from PaVE, and all analyses and figures 1-3 include those HPV strains.
4.  Resolution of the new figures has been improved.
5.  The legend to Figure 2B has been modified as suggested.
6. The legend to Figure 2C describes all symbols and colored blocks.
7. Figure 3A has been updated to show the motifs clearly and arrange the strains by the order in the phylogenetic tree.
8. Figure 3B has been updated, confusing labelling of 18S rRNA removed.

# Response

**Published:** 2019-09-13

**Authors:** Eugene Koonin

**Affiliations:** National Center for Biotechnology Information (NCBI), National Institutes of Health (NIH), USA

Although the referee has suggested a statistical review of the permutation test that was used in this work to assess the significance of the results, we are confident that this is unnecessary because the statistical technique we used is simple and standard.
